# Targeting Protein Kinase G to Treat Cardiac Proteotoxicity

**DOI:** 10.3389/fphys.2020.00858

**Published:** 2020-07-28

**Authors:** Christian U. Oeing, Sumita Mishra, Brittany L. Dunkerly-Eyring, Mark J. Ranek

**Affiliations:** ^1^Division of Cardiology, Department of Medicine, The Johns Hopkins Medical Institutions, Baltimore, MD, United States; ^2^Department of Cardiology, Charité – University Medicine Berlin, Campus Virchow Klinikum (CVK), Berlin, Germany; ^3^DZHK (German Centre for Cardiovascular Research), Partner Site Berlin, Berlin, Germany

**Keywords:** proteostasis, PKG, proteotoxicity, proteasome, autophagy, heart failure

## Abstract

Impaired or insufficient protein kinase G (PKG) signaling and protein quality control (PQC) are hallmarks of most forms of cardiac disease, including heart failure. Their dysregulation has been shown to contribute to and exacerbate cardiac hypertrophy and remodeling, reduced cell survival and disease pathogenesis. Enhancement of PKG signaling and PQC are associated with improved cardiac function and survival in many pre-clinical models of heart disease. While many clinically used pharmacological approaches exist to stimulate PKG, there are no FDA-approved therapies to safely enhance cardiomyocyte PQC. The latter is predominantly due to our lack of knowledge and identification of proteins regulating cardiomyocyte PQC. Recently, multiple studies have demonstrated that PKG regulates PQC in the heart, both during physiological and pathological states. These studies tested already FDA-approved pharmacological therapies to activate PKG, which enhanced cardiomyocyte PQC and alleviated cardiac disease. This review examines the roles of PKG and PQC during disease pathogenesis and summarizes the experimental and clinical data supporting the utility of stimulating PKG to target cardiac proteotoxicity.

## Introduction

Protein kinase G (PKG) elicits cardioprotection during various forms of cardiac stress by transducing a vast array of beneficial processes (Dunkerly-Eyring and Kass, 2019; Pinilla-Vera et al., 2019). PKG stimulates left ventricular relaxation and counters pathological hypertrophy and remodeling (Dunkerly-Eyring and Kass, 2019). Insufficient PKG signaling has been implicated in the pathogenesis of cardiac disease toward heart failure, giving considerable interest to strategies to enhance PKG signaling (Kokkonen and Kass, 2017; Dunkerly-Eyring and Kass, 2019). New pharmacological approaches to stimulate PKG are being evaluated as therapy for heart failure and other forms of cardiac disease in clinical trials (Pinilla-Vera et al., 2019). However, a better understanding of the substrates targeted by PKG for cardioprotection is needed. Recently the activation of PKG was demonstrated to regulate protein homeostasis (proteostasis), to attenuate disease pathogenesis (Dunkerly-Eyring and Kass, 2019). The protein targets, underlying mechanisms, and therapeutic strategies to facilitate PKG regulation of proteostasis are only beginning to be identified.

Cardiomyocyte proteostasis is maintained by elaborate protein quality control (PQC) systems. These systems help fold polypeptide chains into properly functioning proteins, refold proteins that become misfolded or damaged during stress, and then remove terminally misfolded and/or aggregated proteins by degradation (Wang and Robbins, 2006; Wang et al., 2008). Cardiomyocyte PQC is maintained by three separate but interlinking systems: molecular chaperones (protein folding/refolding), the ubiquitin proteasome system (UPS) (proteasome mediated degradation of soluble proteins), and autophagy (lysosomal degradation of protein aggregates and organelles) (Wang et al., 2008). Similar to PKG signaling, cardiomyocyte PQC is also perturbed/insufficient during cardiac disease pathogenesis. This results in the intracellular accumulation of proteins targeted for degradation, aggregation of proteins, and subsequent declined cardiac function; a class of disorders termed cardiac proteotoxicity (Wang and Robbins, 2006; Willis and Patterson, 2013). The proteins and macromolecular structures that are responsible for maintaining cardiomyocyte PQC are known; however, only recently have we begun to understand the proteins and/or posttranslational modifications that regulate PQC. Pharmacological strategies to safely enhance PQC are beginning to be explored. This review focuses on one protein, PKG, which has shown promise as a cardiac PQC-enhancing therapy. We will detail the studies that demonstrate a role for PKG regulating PQC during physiological and pathological states, examine the therapeutic potential of targeting PKG, and discuss potential future directions.

## Proteotoxicity in Heart Failure

The ubiquitin-proteasome system (UPS) removes damaged and/or misfolded proteins that are first tagged by ubiquitination for degradation by the proteasome (Wang et al., 2008). Ubiquitination is mediated by multiprotein ubiquitin-activating enzymes (E1), ubiquitin-conjugating enzymes (E2), and ubiquitin ligases (E3). Ubiquitinated proteins are then translocated to the core proteasome for degradation (Amerik and Hochstrasser, 2004; Reyes-Turcu et al., 2009; Metzger et al., 2012). The mammalian proteasome is a multi-subunit protease complex composed of the 20S catalytic core particle and two 19S regulatory cap particles. The 19S regulatory subunits recognize the polyubiquitin chain and unfold the protein for subsequent degradation by the 20S catalytic core (Nussbaum et al., 1998; Lasker et al., 2012).

All forms of cardiac disease, including heart failure, present with an accumulation of ubiquitinated proteins, demonstrating the vital role the UPS has during pathogenesis (Drews et al., 2010; Su and Wang, 2010; Willis et al., 2010; Wang et al., 2011; Powell et al., 2012; Day, 2013). Indeed, dysfunctional UPS has been reported in human end-stage heart failure, ischemic heart disease, and cardiac hypertrophy (Hein et al., 2003; Weekes et al., 2003; Drews et al., 2010; Powell and Divald, 2010; Predmore et al., 2010). Weekes et al. first reported increased levels of ubiquitinated proteins in myocardial samples obtained from patients with familial dilated cardiomyopathies, which has been supported by others (Weekes et al., 2003; Chen et al., 2005; Liu et al., 2006a, b). Left ventricular unloading in humans with chronic heart failure leads to improved proteasome activity (Wohlschlaeger et al., 2010). Similar results were reported by Predmore et al. (2010), who demonstrated markedly reduced proteolytic activities in failing human hearts that was restored after LV unloading. The mechanism by which ventricular unloading stimulates proteasome activity remains unknown; however, there are multiple potential explanations: (1) LV unloading decreases intracellular ROS, thus oxidized proteins for proteasomal degradation along with less oxidation of the proteasome itself; (2) a separate post-translational modification(s) of the UPS by yet to be identified kinase(s) (Predmore et al., 2010). Proteasome inhibition in pre-clinical models is associated with exacerbated or accelerated pathogenesis of cardiac disease. Mice treated with bortezomib (proteasome inhibitor) worsened transaortic constriction (TAC)-induced cardiac hypertrophy, resulting in early heart failure and death in mice (Tang et al., 2010). These findings were supported by a study using a genetic cardiomyocyte restricted-proteasome inhibited mouse model (beta5^T60A^) following TAC surgery (Ranek et al., 2015). Similarly, models of myocardial ischemia demonstrated that pretreatment of isolated rat hearts with a proteasome inhibitor dose-dependently decreased post-ischemic cardiac function (Powell et al., 2005). Tian et al. (2012) reported mice that express a threonine 60 to alanine mutation on the proteasome subunit beta 5 (beta5^T60A^) to reduce the proteolytic activity of the proteasome had worsened myocardial ischemia-reperfusion injury. Impaired or insufficient proteasome activity has been associated with myocarditis (Szalay et al., 2006) and diabetic cardiomyopathy (Li and Wang, 2011). Genetic overexpression of key proteasome subunits enhances proteasome-mediated protein degradation and protects the heart against oxidative stress, proteotoxicity, and myocardial ischemia (Li et al., 2011a, b). Together, these studies demonstrate the prominent role the UPS has in degrading proteins to maintain proteostasis during cardiac disease.

The other primary mediator of cardiomyocyte proteostasis is autophagy, which is comprised of macroautophagy, microautophagy, chaperone-mediated autophagy, and organelle-specific autophagy (e.g., mitophagy) (Ghosh and Pattison, 2018). Macroautophagy involves the formation of an autophagosome which surrounds the cargo for degradation by the lysosome. Microautophagy is the direct engulfment of cellular debris by the lysosome. Chaperone-mediated autophagy degrades proteins containing a KFERQ pentapeptide motif that are translocated into the lysosome via a heat shock cognate 70 (Hsc70) chaperone complex (Dice, 1990). Autophagy is required for the development, differentiation, and function of cardiomyocytes (Nakai et al., 2007; Zhang et al., 2012; Ikeda et al., 2015) and has an important role in cardioprotection (Gustafsson and Gottlieb, 2008; Sciarretta et al., 2014).

Dysregulated autophagic flux is associated with and has been implicated in the pathogenesis of many forms of cardiac disease. Formation of autophagosomes in dilated cardiomyopathy patients has a positive correlation with better prognosis, indicating the protective role of autophagy in heart failure (Saito et al., 2016). Many pre-clinical models have associated reduced autophagy with heart failure and cardiac functional decline (Eisenberg et al., 2016; Shirakabe et al., 2016; Ghosh and Pattison, 2018). Mice with mutations in *MYBPC3* (cardiac myosin-binding protein C) and in *MYH7* (β-myosin heavy) forms of hypertrophic cardiomyopathy (HCM) present with an accumulation of autophagic vacuoles, suggesting impaired autophagic flux (Schlossarek et al., 2012; Song et al., 2014; Carrier et al., 2015; Singh et al., 2017). Further, deletion of Atg5 (autophagy related gene 5), a key protein involved in the extension of the phagophore membrane during autophagic vesicle formation, causes cardiac hypertrophy and left ventricular dysfunction (Nakai et al., 2007; Pattison et al., 2011). In a cardiac proteinopathy model (CryAB^R120G^), cardiac-specific overexpression of Atg7 increased autophagic activity and improved cardiac function (Bhuiyan et al., 2013). Obese mice see the downregulation of several autophagic genes, including Atg7 (Sciarretta et al., 2012). The exact mechanism for the downregulation of Atg7 was not revealed in these studies; however, it is known that proteinopathy and obesity are characterized by increased mammalian (mechanistic) target of rapamycin complex 1 (mTORC1) activity, which suppresses Atg7 expression (Sciarretta et al., 2018). Interestingly the increased expression of Atg7 in the heart prevents the heart from hypertrophying in response to high-fat diet-induced obesity (Tong et al., 2018). Autophagic flux was reduced in both type 1 and 2 diabetic mouse models and in aged mice (Epstein et al., 1989; Lee et al., 1996; Xie et al., 2011; Kanamori et al., 2015; Munasinghe et al., 2016; Nakamura and Sadoshima, 2018).

**FIGURE 1:**
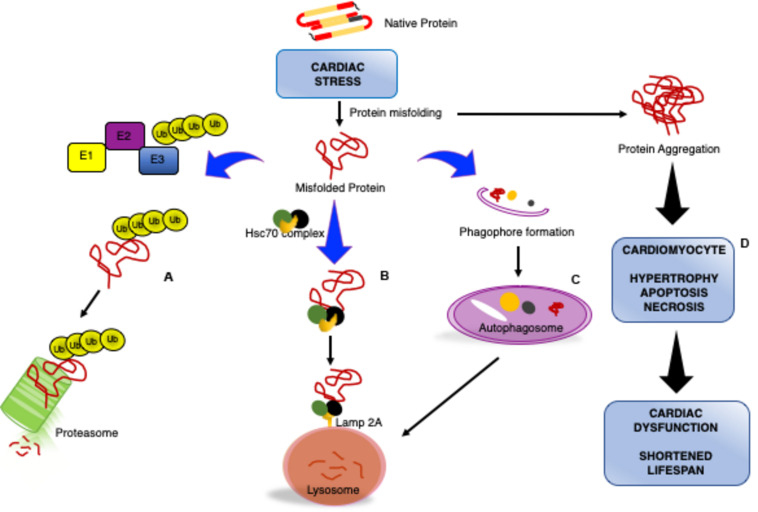
Impaired cardiomyocyte proteostasis results in cardiac dysfunction. Cardiac pathologic stress increases production/formation of misfolded proteins that if not removed form large, insoluble protein aggregates. Cardiomyocytes utilize various processes to maintain proteostasis: misfolded proteins will be catalyzed by the ubiquitin proteasome system (UPS) through ubiquitination via a series of enzymatic reactions involving an ubiquitin activating enzyme (E1), ubiquitin conjugating enzyme (E2), and ubiquitin ligase (E3) for degradation by the proteasome **(A)**. Chaperone-mediated autophagy is a process by which the heat shock cognate 70 (HSC70) complex recognizes and binds select protein targets for internalization and degradation to the lysosome through the lysosome-associated membrane protein 2A (LAMP2A) receptor **(B)**. Macroautophagy is the bulk removal of proteins, protein aggregates, and organelles by first forming an autophagosome to surround the cargo followed by merging with the lysosome for degradation **(C)**. The insufficiency or overwhelming of the protein degradation systems during cardiac stress results in an accumulation of aggregated proteins, culminating in reduced cardiac function and lifespan **(D)**.

Collectively, these studies demonstrate the pivotal role that proteostasis, specifically the UPS and autophagy, has during cardiac pathogenesis (Figure 1). To translate these findings to the clinic to directly target cardiac proteotoxicity, a better understanding of the mechanisms regulating PQC and identification of druggable targets is needed. This review describes exciting investigations into a potential target that has the ability to enhance cardiomyocyte PQC and has many therapeutic strategies available.

### The Role of PKG in Proteotoxicity

Protein kinase G is stimulated by cyclic guanosine monophosphate (cGMP). cGMP is generated downstream of nitric oxide (NO) or natriuretic peptide (NP) activation of guanylate cyclase (GC-1, formerly soluble—sGC) and GC-2 (formerly particulate—pGC), respectively (Dunkerly-Eyring and Kass, 2019). Phosphodiesterases (PDEs) selective for cGMP (PDEs 5 and 9 in the heart) negatively regulate PKG activity. PDE regulation of PKG activity is known to be highly compartmentalized within the cardiomyocyte, adding a level of regulation (Kokkonen and Kass, 2017; Dunkerly-Eyring and Kass, 2019). Indeed, PDE5A primarily regulates NO-generated cGMP, which tends to be dispersed throughout the cytosol of the cardiomyocyte, whereas PDE9A regulates the cGMP pool that is localized at the membrane (Kokkonen and Kass, 2017). PDE1, a dual cAMP and cGMP esterase, regulates PKA and PKG activity in an isoform-specific manner in the heart (Hashimoto et al., 2018; Kokkonen-Simon et al., 2018; Dunkerly-Eyring and Kass, 2019).

Multiple strategies of PKG activation have proven to be cardioprotective in response to various pathological stimuli: inhibition of PDE5 or PDE9 reduced cardiac hypertrophy and improved function following left ventricular pressure overload (PO) induced by TAC (Takimoto et al., 2005; Nagayama et al., 2009; Lee et al., 2015). Stimulating PKG directly reduced infarct size and myocardial fibrosis/remodeling following myocardial infarction and ischemia-reperfusion injury (Das et al., 2006; Salloum et al., 2008; Krieg et al., 2009). Neurohormonal stimulation of G-protein-coupled (Gq and Gi) receptor signaling is suppressed by PKG phosphorylation of and/or binding to the regulator of G protein signaling (RGS) proteins, RGS2 and RGS4 (Takimoto et al., 2009). PKG phosphorylates and inhibits the transient receptor potential cation channels type 6 (TRPC6) to block calcineurin/NFAT signaling (Kinoshita et al., 2010; Koitabashi et al., 2010; Nishida et al., 2010) and RhoA to decrease Rho-kinase (Sawada et al., 2001). PKG also regulates mechanosensing via phosphorylation of the sarcomeric proteins: myosin-binding protein C, phospholamban, TnI, and titin (Raeymaekers et al., 1988; Kruger et al., 2009; Thoonen et al., 2015; Rainer and Kass, 2016). Collectively, these studies demonstrate the ability of PKG to correct pathological imbalances, but evidence that PKG stimulation could restore proteostasis during cardiac disease is more recent and forthcoming.

With PKG acting as a brake on many pathological processes, the attention turned to a potential regulation of cardiomyocyte proteostasis. Over the last decade, multiple studies have indicated that PKG activation enhances PQC as a primary mechanism of action to protect the myocardium. Pioneering studies from the Wang lab discovered PKG positively regulates proteasome activity by phosphorylating key proteasome subunits, Rpt6 of the 19S cap and Beta5 of the 20S proteolytic core (Ranek et al., 2013). Intriguingly, activation of PKG pharmacologically (PDE5 inhibitor, sildenafil) or genetically (expression of a constitutively active PKG) in a proteinopathy model (CryAB^R120G^) reduced the accumulation of ubiquitinated proteins and cleared the degradation of misfolded, but not normal, proteins (Ranek et al., 2013). Enhanced PQC with PKG stimulation was associated with reduced cytotoxicity (*in vitro*) and improved cardiac function (*in vivo*) (Ranek et al., 2013). Similarly, VerPlank et al. (2020) reported stimulation PKG with both a PDE5 inhibitor or GC-1 activator enhanced proteasome proteolytic activity, the degradation of short lived-and long lived proteins, and determined a direct relationship between PKG and purified proteasomes. Stimulation of PKG via activation of the muscarinic 2 receptor also increased proteasome activity (Ranek et al., 2014), suggesting both the plasma membrane localized and cytosolic localized pools of PKG can enhance proteasome activity. Inhibition of PKG or antagonizing muscarinic 2 receptors decreased the proteasome peptidase activities in both the absence or presence of ATP (Ranek et al., 2013, 2014), suggesting that PKG basally regulates proteasome peptidase activities. These data were supported by and expanded on by a recent study using a PDE1 inhibitor, IC86430 (Zhang et al., 2019). Here the authors utilized CryAB^R120G^ proteinopathy mice, which develop a heart failure with a preserved ejection fraction (HFpEF)-like phenotype, and show increased expression of the PDE1A isoform. Inhibition of PDE1 in these mice and cultured cardiomyocytes attenuated proteotoxic stress, increased proteasome activity, and extended mouse lifespan in a PKA and PKG-dependent manner (Zhang et al., 2019). Collectively these findings demonstrate that PKG regulates proteasome activities, proteasome-mediated degradation of misfolded proteins, and that pharmacological approaches can be utilized to elicit these responses.

Recently, it was reported that PKG can also enhance macroautophagy to enhance cardiomyocyte PQC to attenuate cardiac hypertrophy. Tuberous sclerosis complex 2 (TSC2, tuberin), an upstream negative regulator of mTORC1, is phosphorylated by various kinases that can either inhibit (Akt and ERK) or stimulate (AMPK and GSK-3β) its activity. The Kass lab recently reported a new signaling paradigm whereby PKG phosphorylates TSC2 at serine 1365 (1364 in humans) (Ranek et al., 2019). Interestingly, this regulation is itself dependent on the redox state of PKG with reduced phosphorylation of TSC2 detected with oxidation of PKG at cysteine 42 (Oeing et al., 2020). Unlike other TSC2 phosphorylation sites, the phosphorylation of 1365 did not affect basal mTORC1 activity. However, a potent inhibition of mTORC1 hyperactivity was observed once mouse hearts were subjected to hemodynamic (left ventricular pressure overload), or cardiomyocytes to hormonal, stress (endothelin-1). PQC was enhanced as evidenced by upregulation of autophagic flux, clearance of ubiquitinated proteins, and decreased protein aggregation (Ranek et al., 2019). TSC2 S1365 phospho-mimetic decreased, whereas phospho-null exacerbated cardiomyocyte cell size and cytotoxicity following endothelin-1 treatment. Phospho-mimetic mice had attenuated cardiac hypertrophy, improved function, and extended lifespan in response to pressure overload, with opposing findings yielded in phospho-null mice (Ranek et al., 2019). This phosphosite on TSC2 is unique in that there only appears to be mTORC1 regulation in the presence of a pathological co-stimulus, thereby not altering the physiological homeostatic role of mTORC1 (Manning, 2019). Considering the issues with chronic, broad mTORC1 inhibition, utilizing a pharmacological approach with a PKG stimulator represents a unique advantage.

## Therapeutic Strategies to Target Proteotoxicity via PKG

Currently, there are no approved therapeutic strategies to enhance PQC. However, interest has increased as more and more studies implicate exacerbated disease pathogenesis with proteotoxicity, and the evidence in pre-clinical models that PQC enhancement strategies elicit cardioprotection. PKG is an attractive therapeutic target for multiple reasons: (1) PKG modulators have been used in clinic for decades, (2) PKG activators/stimulators boast an excellent safety profile and are well tolerated, and (3) there are many proteins available to interrogate the PKG pathway (Kokkonen-Simon et al., 2018; Dunkerly-Eyring and Kass, 2019; Pinilla-Vera et al., 2019). The first PKG activators were used in the 1800 s in the form of inhaled organic nitrates to treat angina pectoris (Kots et al., 2009; Daiber and Munzel, 2015). It would be roughly 100 years before it was discovered that these work by releasing NO and enhancing cGMP levels (Schlossmann and Schinner, 2012). Therapeutic strategies available to activate the PKG pathway either aim to promote cGMP synthesis (e.g., GC-1 stimulators and activators) or to inhibit cGMP degradation (e.g., PDE5 inhibitors), or both. The GC-1 stimulator, Riociguat, is approved for the treatment of pulmonary arterial hypertension (PAH) as well as inoperable chronic thromboembolic pulmonary hypertension. GC-1 stimulators are being tested in heart failure, diabetic nephropathy, systemic sclerosis, as well as sickle cell disease and central nervous system disease (Xiao et al., 2019). The GC-1 stimulator, vericiguat, was tested in clinical trials for both heart failure with a reduced ejection fraction (HFrEF) [SOCRATES-REDUCED NCT01951625 (Gheorghiade et al., 2015) and VICTORIA NCT02861534 (Armstrong et al., 2018)] and in HFpEF (SOCRATES-PRESERVE NCT01951638) (Filippatos et al., 2017; Pieske et al., 2017). Sacubitril/valsartan (entresto) that combines a neprilysin inhibitor with an angiotensin receptor blocker is increasingly popular as a heart failure therapy, as demonstrated in the PARADIGM-HF (NCT01035255) trial (McMurray et al., 2014; Fala, 2015). The ability of neprilysin inhibitors to increase the levels of natriuretic peptides make PKG a potential target of sacubitril/valsartan (Yan et al., 2003; Riddell and Vader, 2017). Indeed, a 2019 study determined that sacubitril/valsartan decreased cardiac fibrosis in a mouse cardiac hypertrophy model and protected cardiac fibroblasts from myofibroblast transition via PKG-dependent inhibition of RhoA activation (Burke et al., 2019).

These trials did not specifically implicate impairment in proteostasis; however, existing data from human heart tissue suggest that proteostasis is impaired in a disease specific manner. Understanding the PQC systems that are perturbed in different diseases will allow for selective therapeutic targeting. Polyubiquitinated proteins are increased in failing hearts in early as well as late stage disease, suggesting that accumulation of polyubiquitinated proteins occurs before the development of decompensated heart failure (Day, 2013). A histological study from human failing hearts due to idiopathic dilated cardiomyopathy (DCM) noted colocalization of ubiquitin with monodansylcadaverine, a specific marker for autophagic vacuoles, suggesting a link between ubiquitin conjugate accumulation and autophagy (Kostin et al., 2003). Patients with ischemic cardiomyopathy (ICM) and DCM show differential UPS activity patterns. Human ICM heart tissue exhibits reduced trypsin-like proteasomal activity compared to DCM, while both chymotrypsin- and caspase-like proteasomal activities were reduced in DCM and ICM hearts compared to non-failing controls (Spanig et al., 2019). HCM is also characterized by a reduction in chymotrypsin- and caspase-like activities compared to control hearts (Predmore et al., 2010). Failing human hearts exhibit reduced proteasome activity compared to donor controls, which is thought to be related to reduced docking of the 19S proteasome to the 20S proteasome and decreased phosphorylation of Rpt6 (Day, 2013), a potential target of PKG (Ranek et al., 2013). Right ventricular heart disease has not been focused on in clinical trials regarding proteostasis despite emerging data of its important role (Rajagopalan et al., 2013; Drews, 2014; Drews and Taegtmeyer, 2014). Hence, new insights into the regulation of proteostasis via PKG signaling in these diseases might help select the correct patient cohort for successful therapy.

**FIGURE 2:**
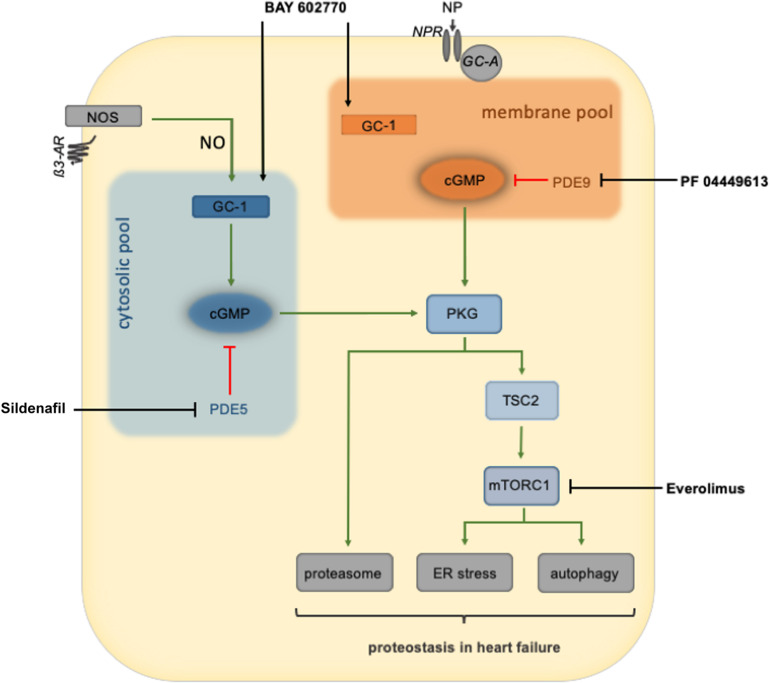
An overview of PKG regulation of cardiomyocyte protein quality control and therapeutic interventions to stimulate PKG activity. PKG is thought to be divided into two primary pools: the membrane and cytosolic pools. Natriuretic peptides binding to the natriuretic peptide receptor activate guanylate cyclase to produce cGMP and ultimately stimulate the membrane pool of PKG. Nitric oxide, produced by nitric oxide synthase, activates guanylate cyclase-1 to produce cGMP, culminating in the activation of the cytosolic PKG pool. Once activated, PKG can increase the activity of the proteasome or phosphorylation of TSC2 to inhibit mTORC1 and enhance autophagic flux. PKG stimulation of the proteasome or autophagy restores cardiomyocyte proteostasis during cardiac stress. AR, adrenoreceptor; GC-1, soluble guanylate cyclase 1; GC-A, guanylyl cyclase-A (*receptor*); GMP, cyclic guanylyl monophosphate; ER, endoplasmatic reticulum; mTORC1, mammalian target of rapamycin complex 1; NOS, nitric oxide synthase; NP, natriuretic peptide; NPR, NP receptor; PDE, phosphodiesterase; PKG, protein kinase G; PQC, protein quality control; TSC2, tuberin/tuberous sclerosis complex 2.

Although the PKG pathway has long been a focus to treat cardiac disease, only recently has the stimulation of PKG been suggested as a new therapeutic strategy to treat cardiac proteinopathies (Figure 2). HFpEF is a lethal syndrome for which there are no evidence-based therapies, characterized by an imbalance in NO levels and low myocardial cGMP and PKG activity (Rainer and Kass, 2016; Schiattarella et al., 2019). A novel murine HFpEF model required metabolic stress accompanied by L-NAME, an NO synthase inhibitor, hence PKG inhibitor, to present some HFpEF symptoms (Schiattarella et al., 2019). These findings suggest that decreased PKG activity facilitates the development and pathogenesis of HFpEF. PDE5 inhibition reduced ER stress in isoproterenol-treated rats and pressure-overloaded mice (Rainer and Kass, 2016) and could successfully treat a desmin-related proteinopathy of the murine heart (Ranek et al., 2013). Xuejun Wang’s group reported that inhibiting PDE1, which hydrolyzes both cAMP and cGMP, increases 26S proteasome activity in a CryAB^R120G^-based proteinopathy of the murine heart (Zhang et al., 2019). Treatment with PDE1 inhibitor IC86430 increased proteasome phosphorylation, reduced misfolded CryAB protein in the murine heart, attenuated HFpEF-like phenotype, and ultimately improved survival (Zhang et al., 2019). These studies further support the notion that activating PKG could be beneficial in HFpEF therapy, at least in part by enhancing PQC. Inhibitors of mTORC1 potently increase autophagic flux, attenuate cardiac hypertrophy, and enhance function; however, chronic use leads to cardiac dilation and failure along with immunosuppression and metabolic disturbances (Benjamin et al., 2011; Zeng et al., 2013). These deleterious side effects can be avoided by stimulating PKG to inhibit mTORC1, hence might present a better tool than mTOR inhibitors (Manning, 2019; Ranek et al., 2019). Collectively these studies demonstrate that (1) PKG is vital to maintain proteostasis basally, (2) many therapeutic targets (NO, NPs, PDEs) are available to stimulate PKG, and (3) PKG activators/stimulators could be the first therapy that enhances PQC, is cardioprotective, and does so without deleterious side effects.

## Conclusion

Our knowledge of the protein kinases that regulate cardiomyocyte PQC has vastly expanded. The discovery of new targets to pursue, pharmacological strategies to test, and increased understanding of the regulatory mechanisms are pivotal to translating successful experimental studies into efficacious clinical therapies. Several crucial hurdles remain. The first being the safety of a therapy, as agents may increase PQC but as a compensatory mechanism for damage induced by the therapy. Second issue is having druggable targets for therapeutic interventions. The third hurdle is to match the appropriate disease state to the PQC deficiency to the microdomain in which the kinase and PQC system reside. PKG activators/stimulators are safe and well tolerated and many are already in clinical use. Further research to gain a precise understanding of the microdomains these compounds work in and PQC machinery they associate with will identify the conditions and patient subsets that will likely benefit from specific PKG modulation. The final hurdle is the need for a blood biomarker capable of detecting impaired proteostasis in the heart, which to the best of our knowledge has not been verified. This would be essential to detect patients that might benefit from strategies to improve cardiomyocyte PQC and to monitor potential success of the therapy.

## Disclosure Statement

BD-E and MR are co-inventors on a patent application (PCT: 448070145WO1) that was filed in July 2018 (provisional filed in June 2017). The patent relates to the use of TSC2(S1365/S1364) modifications for immunological applications.

## Author Contributions

MR conceived and designed the framework for the review and supervised the writing. CO wrote the manuscript with assistance from SM and BD-E. All authors provided critical feedback while writing the manuscript.

## Conflict of Interest

MR is a co-founder and shareholder of Meta-T Cellular, a start-up company that aims to develop applications of this intellectual property for immune therapy.

The remaining authors declare that the research was conducted in the absence of any commercial or financial relationships that could be construed as a potential conflict of interest.
